# Oxidative Stress in Wheat Caused by Ampicillin and Amoxicillin and Their Mixture Applied to the Soil

**DOI:** 10.3390/ijms26178156

**Published:** 2025-08-22

**Authors:** Robert Biczak, Arkadiusz Telesiński, Marcin Sysa, Agnieszka Godela, Barbara Pawłowska

**Affiliations:** 1 The Faculty of Science and Technology, Jan Długosz University in Czestochowa, 13/15 Armii Krajowej Av., 42-200 Czestochowa, Poland; r.biczak@ujd.edu.pl (R.B.); m.sysa@ujd.edu.pl (M.S.); a.godela@ujd.edu.pl (A.G.); 2 The Faculty of Environmental Management and Agriculture, West Pomeranian University of Technology, Juliusza Słowackiego st. 17, 71-434 Szczecin, Poland; arkadiusz.telesinski@zut.edu.pl

**Keywords:** antioxidant enzyme activity, chlorophyll fluorescence, oxidative stress, photosynthetic pigments, antibiotics

## Abstract

Ampicillin (AMP) and amoxicillin (AMX) are widely used penicillin antibiotics. After administration to humans and animals, they are largely excreted in unchanged or metabolized forms, leading to their release into wastewater. In surface waters, their concentrations usually reach the ng∙L^−1^ range and rarely exceed µg∙L^−1^, although in India AMX levels above mg∙L^−1^ were detected in hospital effluents. The limited efficiency of wastewater treatment plants allows these compounds to enter aquatic and terrestrial environments, where they affect various organisms. The aim of this study was to assess the effects of AMP, AMX, and their mixture on wheat, one of the most extensively cultivated cereals. Determinations were carried out using standardized methodologies. The results showed that antibiotics induce oxidative stress in plants, with symptoms observed only at concentrations of 1000 mg∙kg^−1^ of soil DW. At this level, changes included altered antioxidant enzyme activity (APX, SOD, POD, and CAT), increased proline and H_2_O_2_ content, and reduced MDA levels. By contrast, antibiotics had minimal influence on glutathione and ascorbate and caused only slight changes in photosynthetic pigments and chlorophyll fluorescence.

## 1. Introduction

Antibiotics are a major group of pharmaceuticals used against bacterial infections in humans and animals and applied to enhance livestock productivity [1]. Like other pharmaceuticals and personal care products (PPCPs), antibiotics pose environmental risks due to their widespread use. Large amounts are excreted unmetabolized in urine and feces, with up to 75% of veterinary antibiotics entering the environment directly, a phenomenon also noted for human drugs [2]. Owing to their continuous release, they are considered “pseudo-persistent” pollutants [1]. Most human-used antibiotics reach wastewater and cannot be efficiently removed by conventional treatment plants, leading to their accumulation in sewage sludge and treated effluents and detection in aquatic ecosystems worldwide. Antibiotics also enter soils via sludge fertilizers, irrigation with treated wastewater (common in arid regions), animal manure, landfill leachates, or groundwater infiltration. In soil and water, parent compounds and metabolites undergo biological, chemical, and physical transformations, while the extent of contamination depends on soil properties (e.g., organic matter, pH) and compound physicochemistry [3,4].

Pharmaceuticals, including antibiotics, threaten food safety and environmental health. Soil contamination alters microbial communities and activity, while plants absorb antibiotics, which can trigger oxidative stress and impair development. These effects result from disruption of DNA replication, transcription, and metabolism, with consequences for ecological safety [2,5,6]. Reported plant effects include reduced germination, ROS overproduction, cell damage, inhibited root elongation, and biomass loss. Moreover, antibiotic persistence fosters resistant bacteria, increasing risks to human and animal health via the food chain [6,7,8].

Thus, comprehensive assessment of drug impacts on plants is essential, as compounds may act additively, antagonistically, or synergistically [9]. Plants counter ROS accumulation through antioxidant systems (AOX) comprising non-enzymatic components (carotenoids, flavonoids, proline, glutathione, and ascorbate) and enzymatic defenses (CAT, GPX, APX, and SOD) [3].

Yet, mechanisms of antibiotic action on plants remain poorly understood [1]. It is known that xenobiotic-induced oxidative stress causes ROS overproduction, leading to lipid peroxidation, nucleic acid and protein damage, enzyme inhibition, and even cell death [3].

This study therefore examined the effects of ampicillin (AMP) and amoxicillin (AMX) on oxidative stress in seedlings of wheat, one of the world’s most important cereals. Since AMP and AMX often occur as mixtures, their combined impact was also investigated. To our knowledge, this is the first study comparing AMX, AMP, and their binary mixtures in wheat.

AMP and AMX were selected as widely used penicillin-group antibiotics administered to both humans and animals [10]. Up to 80–90% of amoxicillin is excreted unchanged, making it a notable environmental hazard [10,11].

To assess their effects, we measured chlorophyll fluorescence, phtotosynthetic pigment content, antioxidant enzyme activity (SOD, POD, CAT, APX, and GPX), and concentrations of proline, MDA, H_2_O_2_, AsA, and GSH.

## 2. Results and Discussion

### 2.1. Photosynthetic Pigments

All green plants have photosynthetic pigments, such as chlorophyll and carotenoids, which are essential for photosynthesis. Photosynthetic pigments absorb light energy at specific wavelengths and reflect others. Chlorophyll a also converts light into chemical energy, while carotenoids assist in light capture and protect plants by dissipating excess energy as heat. The content of photosynthetic pigments is therefore a key parameter for assessing the influence of environmental factors on plants [12,13].

In this study, exposure to AMP, AMX, and their mixture slightly increased photosynthetic pigment content in wheat seedlings at low concentrations (0.1–1 mg·kg^−1^ of soil DW), possibly reflecting hormesis. At higher concentrations (10–1000 mg·kg^−1^ of soil DW), photosynthetic pigment levels declined to the control or below. Antibiotic exposure also caused a slight reduction in the chlorophyll a/b ratio (chla/chlb), indicating adaptive adjustments in light-harvesting complexes (LHCs) and possible weakening of photosynthetic capacity since chlorophyll a is directly involved in photochemistry. Conversely, AMX and the AMP + AMX mixture increased the total chlorophyll/carotenoid ratio, while AMP alone first decreased and then slightly increased it (Table 1). These changes suggest oxidative stress but also adaptive responses in wheat seedlings.

According to literature reports [4,9,14] plant responses to drugs vary. Xiong et al. [13,15] and Gomaa et al. [9] found that pharmaceuticals can stimulate photosynthetic pigment content in microalgae as a defense against ROS accumulation. In contrast, Taschina et al. [4], Opriş et al. [1], and Baciak et al. [16] reported photosynthetic pigment reduction in plants, likely due to drug-induced cellular disorders suppressing photosynthetic pigment synthesis, resulting in lower photosynthetic activity and carbon uptake. Alkimin et al. [14] demonstrate that drugs effects are not always visible in photosynthetic pigment content, proposing two explanations: plant acclimatization over time or insufficient drug concentration to disrupt photosynthetic pigment synthesis.

It should also be noted that the present study focused only on the early growth stage of wheat (14 days after sowing), leaving long-term effects unknown.

### 2.2. Chlorophyll Fluorescence

In addition to photosynthetic pigment content, chlorophyll fluorescence is key parameter for assessing stress effects on photosynthesis. The method is valued for its speed, precision, and non-invasive application [17,18]. Photosynthetic pigments, such as chlorophylls and carotenoids, in antenna complexes absorb solar energy, which is transferred to PSI and PSII for photochemical reactions. Part of the energy is dissipated as heat, while another fraction is re-emitted as fluorescence. About 90% of the signal originates from chlorophyll a in PSII since excitation energy from chlorophyll b is transferred to chlorophyll a. Fluorescence from PSI is minimal and masked at room temperature, so in vivo fluorescence reflects mainly PSII activity [18,19,20].

The recorded parameters include initial (zero) fluorescence (F_0_), maximum fluorescence after dark adaptation (F_m_), variable fluorescence (F_v_), the maximum PSII photochemical yield (F_v_/F_m_), PSII electron yield, and the photosynthetic quantum conversion factor (F_v_/F_0_).

Neither AMP nor AMX alone altered fluorescence in wheat seedlings, independent of concentration. In contrast, the AMP + AMX mixture reduced F_0_, F_m_, and F_v_ while slightly increasing F_v_/F_m_ and F_v_/F_0_ (Table 2). Thus, despite reductions in absolute values, the rise in relative indicators suggests PSII was not directly damaged. Combined with photosynthetic pigment content data, the results indicate activation of adaptive mechanisms, likely involving adjustments in antenna composition and photosynthetic efficiency.

Although individual antibiotics showed no effect, their combination did, highlighting risks since pharmaceuticals often co-occur in the environment. It should be noted that measurements were performed 14 days after sowing, i.e., during early wheat growth. The lack of changes at this stage does not preclude later effects, and observed responses to AMP + AMX mixture may reduce yield in later development.

According to the available literature [19,21,22] algal species differ in sensitivity to pharmaceuticals. For example, AMX strongly reduced fluorescence in *Synechocystis* sp. at concentrations ≥ 1 mg·L^−1^, whereas *Alexandrium tamarense* showed no sensitivity to AMP. Similar variability is expected in higher plants.

Increases in F_0_ indicate greater excitation loss in antenna–reaction center transfer. Rising F_0_ and F_m_ may signal reaction center damage, while a decline in F_v_/F_m_ reflects PSII impairment through lower quantum yield and higher heat dissipation [23].

### 2.3. Ascorbic Acid (AsA) and Glutathione (GSH)

Glutathione and ascorbate are key small-molecule antioxidants active in the Halliwell–Asada cycle, which removes reactive oxygen species (ROS) and protects plant cells from oxidative stress. Ascorbate scavenges free radicals, regenerates tocopheroxyl radicals, and acts as a cofactor for ROS-detoxifying enzymes. Glutathione, although not water-soluble like ascorbate, protects cell membranes where ascorbate activity is limited [8,24].

**Figure 1:**
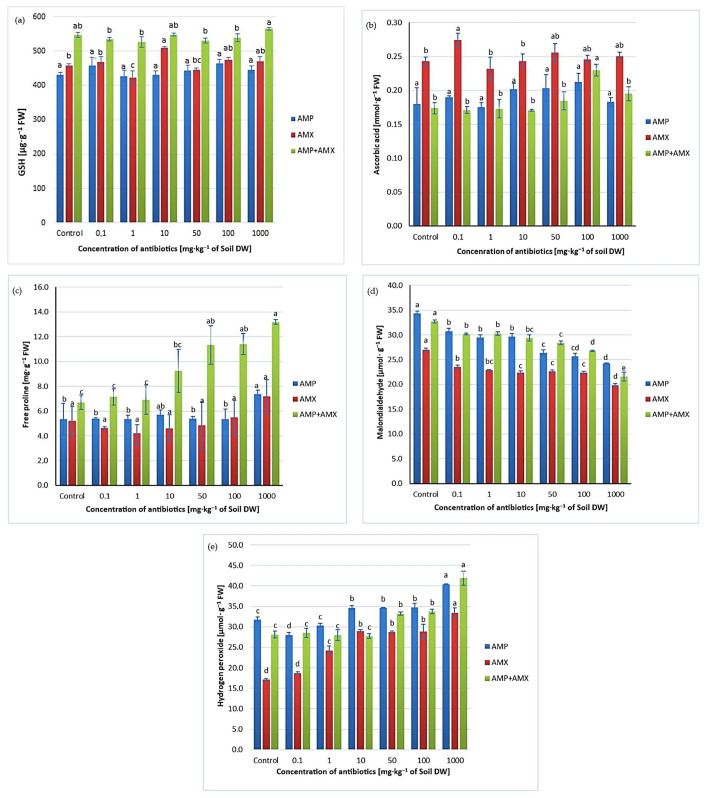
Contents of GSH (**a**), ASA (**b**), free proline (**c**), MDA (**d**), and H_2_O_2_ (**e**) in wheat seedlings growing on soils contaminated with AMP, AMX, and a mixture of AMP + AMX. Data are means ± SD from 3 independent experiments. Values denoted by the same letters for the same biomarkers do not differ statistically at *p* < 0.05.

The study revealed only minor, concentration-independent changes in glutathione (Figure 1a) and ascorbate (Figure 1b). Responses differed between individual antibiotics and their mixture, suggesting weak or transient effects on antioxidant metabolism, possibly masked by soil properties or microbial activity. This variability underlines the complexity of phytotoxic mechanisms, involving both direct plant effects and indirect interactions with microflora. Observed changes may also reflect adaptive redox balance mechanism buffering stress.

In plants, glutathione exists in oxidized (GSSG) and reduced (GSH) forms, which interconvert to maintain homeostasis. Drugs can conjugate with GSH, depleting reduced GSH and producing GSSG. Thus, the GSH/GSSG ratio is a crucial defense marker. Declines in GSH may enhance oxidative stress but also enable xenobiotic detoxification. Conversely, the absence of measurable change may indicate rapid GSH consumption during detoxification [7,8].

Ascorbate plays an equally critical role, cooperating with ascorbate peroxidase (APX) to detoxify hydrogen peroxide. Alterations in AsA levels may reflect antibiotic-induced oxidative stress, yet only slight variations were observed. This suggests the antioxidant system remained effective, enabling wheat seedlings to mitigate stress without major growth impairment [24,25].

### 2.4. Free Proline

Proline is a proteinogenic amino acid essential for plant cell protection against stress. Its metabolism affects mitochondrial ROS production and regulates cell survival, though the exact mechanisms remain unclear. Proline acts through several pathways: it accumulates via biosynthesis and functions as an osmolyte or scavenger of ·OH and ^1^O_2_; it also interacts with other metabolic processes, maintaining cellular energy and NADP^+^/NADPH balance by activating survival signaling. In addition, it supports the tricarboxylic acid cycle and glutathione biosynthesis. Accumulation of proline has been documented under stress in bacteria, protozoa, algae, invertebrates, and plants, and concentrations may rise up to 100-fold in stressed plants [3,26,27].

Our study revealed a concentration-dependent increase in proline in wheat cultivated in soil containing AMP, AMX, or AMP + AMX mixtures, with the strongest effect from the mixture (Figure 1c). This suggests that wheat activates defense mechanisms under antibiotic stress, with the AMP + AMX combination producing more pronounced synergistic effects than single compounds.

These findings align with earlier research: Sousa et al. [3] observed proline accumulation in Solanum lycopersicum exposed to diclofenac, and Stuchlíková et al. [28] reported similar increases in Plantago lanceolata with flubendazole and fenbendazole. As with other oxidative stress indicators, proline responses vary with plant species, developmental stage, and stressor type.

### 2.5. Malondialdehyde (MDA) and Hydrogen Peroxide (H_2_O_2_)

Both biotic and abiotic factors can elevate ROS in plant cells, inducing oxidative stress. Hydrogen peroxide, a key ROS, not only signals defense responses but also contributes to lipid peroxidation, measurable by malondialdehyde (MDA) levels [5,8].

In this study, wheat seedlings grown in soil containing AMP, AMX, or AMP + AMX mixtures showed increased H_2_O_2_ (Figure 1e) and reduced MDA (Figure 1d), with stronger effects at higher concentrations. These changes confirm that antibiotics influence plants, yet efficient antioxidant systems prevented visible damage. Thus, antibiotic presence in soil induces mild stress, with H_2_O_2_ acting as a signaling molecule rather than causing structural injury.

Previous studies support these findings: Gomaa et al. [9], Wang et al. [18,29], and Zhang et al. [8] observed increased H_2_O_2_ and MDA in plants and algae exposed to pharmaceuticals. Hassan et al. [30] showed that proline accumulation enhances antioxidant enzyme activity, reducing both H_2_O_2_ and MDA. Similarly, Hnilicková et al. [31] reported that activation of defense pathways and antioxidant systems limits ROS production and lipid peroxidation.

### 2.6. Antioxidant Enzymes

To protect cells from oxidative stress, plants developed enzymatic and non-enzymatic defense systems. The enzymatic system includes superoxide dismutase (SOD), catalase (CAT), peroxidases (POD, APX), and glutathione reductase, which collectively regulate ROS [18,32].

**Figure 2:**
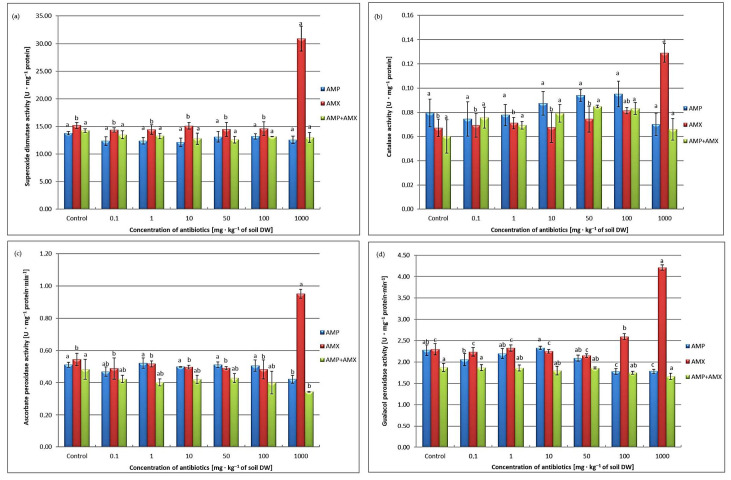
Changes in enzymatic activities of SOD (**a**), CAT (**b**), APX (**c**), and POD (**d**) in the seedlings of wheat treated with AMP, AMX, and AMP + AMX mixtures. Data are means ± SD from 3 independent experiments. Values denoted by the same letters for the same biomarkers do not differ statistically at *p* < 0.05.

SOD, the primary defense enzyme, converts superoxide radicals into H_2_O_2_ and O_2_, limiting lipid peroxidation [7,17,33]. In this study, SOD activity showed no significant changes, except for a >20% increase with AMX at 1000 mg·kg^−1^ soil DW (Figure 2a). The lack of correlation with elevated H_2_O_2_ may reflect other detoxification mechanisms, including the ascorbate redox system and APX activity. Moreover, overall activity may not fully represent isoenzyme diversity, as SOD comprises Fe, Mn, and Cu—metalloenzymes located in different organelles [33,34]. This study also examined only seedlings, not roots, which could show distinct responses.

SOD-generated H_2_O_2_ is subsequently degraded by CAT and peroxidases. CAT decomposes H_2_O_2_ directly, whereas APX and POD require electron donors such as ascorbate or phenolic compounds [2,9,34].

The antibiotics influenced antioxidant enzymes differently. AMP, AMX, and AMP + AMX mixtures initially stimulated CAT, followed by a decline, with a significant increase observed only for AMX at 1000 mg·kg^−1^ of soil DW (Figure 2b). AMP and the mixture slightly reduced APX and POD activity, whereas AMX alone enhanced both (Figure 2c,d).

These results indicate that antibiotics activate enzymatic defenses in wheat seedlings. Elevated APX points to an active ascorbate–glutathione cycle, efficiently removing H_2_O_2_ and preventing cell injury. The absence of visible growth inhibition suggests this system buffers stress effectively. However, high antibiotic concentrations can disrupt antioxidant balance, as shown by the strong response with AMX at 1000 mg·kg^−1^ of soil DW. Thus, plant responses are compound-specific, concentration-dependent, and variable among species, reflecting the complexity of antibiotic-induced oxidative stress. This highlights the need for further research on their ecological and health implications [7,35].

### 2.7. Pearson Correlation

**Figure 3:**
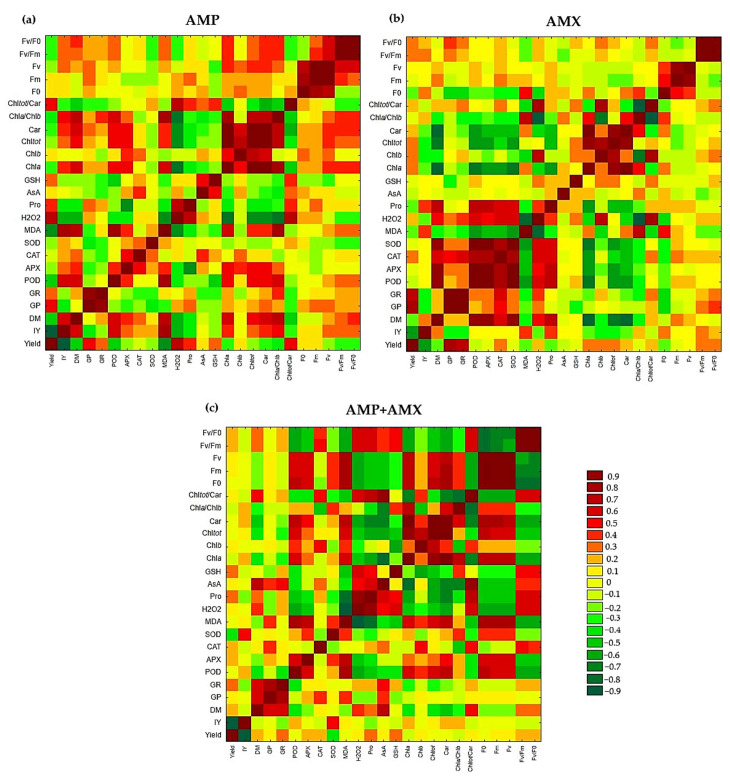
Heat map showing the cluster of Pearson correlation coefficient between individual studied parameters in wheat seedlings exposed to AMP (**a**), AMX (**b**), and AMP + AMX (**c**) mixture. The strength of Pearson correlation coefficients is presented using a green–yellow–red color scheme.

Among plants grown in soil treated with AMX, the strongest positive correlations were observed between Chla and Car (r = 0.99), SOD and POD (r = 0.96), F_v_/F_m_ and F_v_/F_0_ (r = 0.96), GR and GP (r = 0.91), and APX and SOD (r = 0.91). The strongest negative correlations were found between H_2_O_2_ and Chla (r = −0.91), Chla + b/Car and Chla/Chlb (r = −0.91), and H_2_O_2_ and Chla/Chlb (r = −0.91). As indicated by the above correlations, that oxidative stress is correlated with impaired photosynthetic processes in plants exposed to AMX. High levels of MDA and H_2_O_2_ may expert a stronger inhibitory effect on chlorophyll content and fluorescence parameters, ultimately translating into reduced yield (Figure 3b). After AMP application, the strongest positive correlations were recorded between F_v_/F_0_ and Fv/Fm (r = 0.99), Chla + b and Chla (r = 0.97), Car and Chla (r = 0.97), and F_v_ and F_m_ (r = 0.92). The strongest negative correlation was observed between fresh mass yield and yield inhibition (r = −0.99). The observed correlations suggest that photosynthetic mechanisms and the antioxidant response are intensified in wheat seedlings growing in AMP-contaminated soil (Figure 3a). At the same time, oxidative stress correlated negatively with yield, indicating its potential to limit plant growth and productivity. Following the addition of the AMP + AMX mixture to the soil, the strongest positive correlations were found between F_m_ and F_0_ (r = 0.99), F_v_ and F_m_ (r = 0.99), F_v_ and F_0_ (r = 0.98), and Chla and Car (r = 0.93). The strongest negative correlations were observed between fresh mass yield and yield inhibition (r = −0.96) and H_2_O_2_ and MDA (r = −0.93) (Figure 3c). The presence of the AMP + AMX mixture appears to trigger a more complex physiological response. Possible synergy or antagonism between these compounds may produce dispersed effects, meaning that plants do not respond in a uniform way, as reflected by the weaker correlations. This pattern may suggest the activation of compensatory defense mechanisms, which help mitigate stress effects but may also reduce the positive influence of individual factors.

## 3. Materials and Methods

### 3.1. Chemical Reagents

Ampicillin (anhydrous, 96.0–102.0%) and amoxicillin (potency: ≥900 μg per mg) were purchased from Sigma-Aldrich Chemical Co.

### 3.2. Plant Cultivation—How the Research Was Conducted

**Figure 4:**
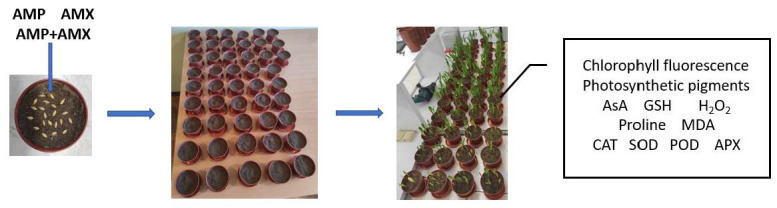
Block diagram of the experiment.

Figure 4 shows a block diagram of the experiment’s execution. The research was conducted in the vegetation hall at Jan Długosz University in Częstochowa. In accordance with the OECD/OCDE 208/2006 guide [36], 20 identical seeds of wheat (*Triticum aestivum* L.) of the Jutrzenka variety purchased from the Breeding and Production Plant in Nieznanice, part of the company Małopolska Hodowla Roślin–HBP Sp. z o.o., Nieznanice, Poland, were sown into pots filled with soil mixed mechanically with AMP, AMX, and AMP + AMX solutions (in a 1:1 ratio). The soil used for testing was air-dried, mechanically homogenized, and then sieved through 2 mm mesh screens. Analysis of the soil’s granulometric composition showed that it was loamy sand, an approximate 11% of the fraction having a diameter of <0.02 mm, 8.5 g·kg^−1^ of organic carbon, and a pH (KCl) equal to 6.7. Antibiotics were introduced into the soil in order to obtain concentrations of 0, 0.1, 1, 10, 100, and 1000 mg·kg^−1^ of soil dry weight (DW) in individual pots. Throughout the study period, the following conditions were maintained in the vegetation hall: temperature 20 ± 2 °C, illumination at 170 µmol∙m^−2^∙s^−1^ with a 16 h day/8 h night regime and soil moisture in the pots −70% field water capacity. All determinations were performed in 3 replicates. Fourteen days after sowing wheat seeds into the soil, basic phytotoxicity parameter determinations were carried out, as described in Pawłowska et al. [37]. Chlorophyll fluorescence was also measured, and material was collected for the subsequent analyses.

### 3.3. Contents of Photosynthetic Pigments

The chlorophyll and carotenoid contents were determined using the methodology proposed by Oren et al. [38]. To do so, 200 mg of plant leaves were homogenized with an 80% acetone solution (4 °C) and then centrifuged at 15,000 rpm for 20 min at 4 °C. The photosynthetic pigment content was determined by measuring the absorbance at 470, 647 and 664 nm wavelengths, followed by calculations using the appropriate formulas:

a = 11.78·A_664_ − 2.29·A_647_ (1)

b = 20.05·A_647_ − 4.77·A_664_ (2)

c = 1000·A_470_ − 3.27·a − 104·b (3)

chl *a* = 25·a/m (4)

chl *b* = 25·b/m (5)

Car = 25·c/(229·m)(6)

where m is the mass of plant material [mg].

All results were converted to dry weight.

### 3.4. Chlorophyll Fluorescence

Chlorophyll fluorescence was determined using an OS1p type chlorophyll fluorescence meter (GEOMOR TECHNIK, Szczecin, Poland). Using the instrument, the following were determined: initial (minimum) fluorescence (F_0_), maximum fluorescence (F_m_), fluctuating fluorescence (F_v_), maximum quantum yield of PSII photochemistry (F_v_/F_m_), and its more sensitive form F_v_/F_0_.

### 3.5. Ascorbic Acid (AsA) and Glutathione (GSH)

To perform the analysis, 0.5 g of fresh wheat plant material was weighed and homogenized with 10% trichloroacetic acid (TCA). The mixture was then centrifuged at 15,000 rpm for 20 min at 4 °C. To determine AsA, a reaction mixture was prepared containing 0.2 mL of the supernatant, 1M H_3_PO_4_, 2% TCA, 0.15% FeCl_3_, and 0.8% bipyridyl and incubated in the dark for 60 min at 37 °C. The absorbance of the mixture was measured at 525 nm. The AsA content was read from a standard curve of ascorbic acid of 0–40 nmol [39].

The GSH content was determined by preparing a reaction mixture containing the supernatant, a phosphate buffer at pH 7.7, and 5,5′-dithiobis(2-nitrobenzoic) acid (DTNB). This mixture was then incubated for 5 min at 30 °C. The absorbance of the reaction mixture was measured at 412 nm [40].

### 3.6. Free Proline

To perform the analysis, 0.5 g of fresh plant material was weighed and homogenized in a solution of 3% sulfosalicylic acid. The mixture was then centrifuged at 15,000 rpm for 20 min at 4 °C. The reaction mixture, which contained 1 mL of the supernatant, 1 mL of acid ninhydrin, and 1 mL of glacial acetic acid, was incubated for 1 h at 100 °C and then placed on ice. After cooling, extraction was performed using toluene. The absorbance of the organic layer was measured at 520 nm. Free proline content was determined using a standard curve [41].

### 3.7. Malondialdehyde (MDA) and Hydrogen Peroxide (H_2_O_2_)

To perform the analysis, 0.5 g of fresh plant material was weighed and homogenized with 0.1% trichloroacetic acid. The mixture was then centrifuged, and the resulting supernatant was used to determine the MDA and H_2_O_2_ levels.

To determine MDA, the reaction mixture containing the supernatant, a phosphate buffer at pH 7.6, and 0.5% thiobarbituric acid (TBA) in 20% trichloroacetic acid was incubated in a water bath at 95 °C for 30 min. Then, the tubes were placed in an ice bath. The absorbance of the reaction mixture was measured at 532 and 600 nm [42]. The MDA concentration was calculated using the following formula:

C_MDA_ = (∆E∙V_0_)/(ε∙V∙g) (7)

where

ΔE = E_532_ − E_600_;

V_0_—total volume of supernatant [mL];

ε—mole absorption coefficient—155 mM·cm^−1^;

V—volume of supernatant added to the reaction mixture [mL];

m—fresh leaf mass [g].

The H_2_O_2_ content was determined by preparing a reaction mixture containing the supernatant and a phosphate buffer at pH 7.6 and 1 M KI. This mixture was incubated in darkness for one hour. The absorbance of the reaction mixture was measured at 390 nm [43]. The H_2_O_2_ concentration was calculated using the following formula:

C = (C_st_ × V_o_)/(V × m) (8)

where

V_o_—total amount of supernatant [mL];

V—volume of supernatant added to the reaction mixture [mL];

m—leaf mass (g).

C_st_ = A/ε × l (9)

where

A—absorbance;

l—thickness of the absorbing layer (1 cm);

ε—molar absorption coefficient—155 mM·cm^−1^.

### 3.8. Antioxidant Enzymes

To perform the analysis, 0.5 g of fresh plant material was weighed and homogenized with an extraction mixture containing phosphate buffer (pH 7.4), 1% polyvinylpyrrolidone (PVP solution), and 1 mM EDTA solution. The mixture was then centrifuged and used to determine antioxidant enzyme activity and protein content.

The total protein content was determined using a colorimetric method based on the reaction of the proteins with Coomassie Brilliant Blue (the Bradford method). [44].

Catalase (CAT) activity was determined performed by mixing the supernatant, a phosphate buffer at pH 7.7, and H_2_O_2_ (30 mmol·L^−1^). Absorbance at 240 nm was measured for 3 min [45].

Ascorbate peroxidase (APX) activity was determined by measuring the absorbance of a reaction mixture containing supernatant, ascorbate (7.5 mM), phosphate buffer (pH 7.0), and hydrogen peroxide (300 mM) at 290 nm for 3 min [46].

Guaiacol peroxidase (POD) activity was determined by measuring the absorbance (at 470 nm) of a reaction mixture containing phosphate buffer (pH 6.0), guaiacol (96 mM), H_2_O_2_ (12 mM), distilled water, and supernatant for 3 min [47].

Superoxide dismutase (SOD) activity was determined spectrophotometrically by measuring the reduction rate of nitrotetrazolium blue (NBT) [48].

### 3.9. Statistical Analysis

The data obtained during the research were statistically analyzed using STATISTICA 13.3. One-way analysis ANOVA was performed, followed by a Tukey post hoc test. All analyses were performed three times (n = 3), and the results are presented as the arithmetic mean ± standard deviation. Significant differences were found at *p* < 0.05. Additionally, a Pearson correlation coefficient analysis was performed to determine the relationship between the various.

Due to the low toxicity of the tested antibiotics, the EC_50_ value could not be determined.

## 4. Conclusions

The results indicate that amoxicillin (AMX), ampicillin (AMP), and their mixture adversely affect wheat, though the alterations were moderate and did not significantly impair early development. Wheat seedlings responded to antibiotics in the substrate through changes in antioxidant enzyme activity, H_2_O_2_, MDA, and proline levels, confirming that these compounds are biologically active. The strongest impact on chlorophyll fluorescence was observed for the AMP + AMX mixture. Since pharmaceuticals in natural environments usually occur in mixtures with other pollutants, the effects of additional co-contaminants may be considerably more severe.

These findings, consistent with other studies, highlight the urgent need for broad environmental assessments of pharmaceuticals, particularly their effects on plants as the basis of food chains. Antibiotics can accumulate in plant tissues, enabling transfer through the food chain, which may threaten human health and facilitate the emergence of drug-resistant bacteria.

It must be stressed that the present experiments addressed only early wheat development. As AMP, AMX, and their mixture influenced growth, future field studies should explore long-term effects on later stages and yield. Moreover, metabolomic and proteomic approaches are recommended to clarify molecular mechanisms of plant responses to antibiotics.

## Figures and Tables

**Table 1:** Effect of AMP, AMX, and AMP + AMX on the photosynthetic pigment in seedlings of wheat. Data are expressed as the mean ± SD of three replicates for each concentration. Values denoted by the same letters in the columns do not differ statistically at *p* < 0.05.

Concentration of Antibiotics (mg·kg^−1^ of Soil DW)	Pigments (mg·g^−1^ DW)					
	Chl*a*	Chl*b*	Chl*a* + Chl*b*	Chl*a*/Chl*b*	Car	Chl(*a* + *b*)/Car
AMP						
0	9.045 ± 0.117 ^c^	4.431 ± 0.113 ^c^	13.476 ± 0.229 ^bcd^	2.042 ± 0.027 ^b^	2.193 ± 0.029 ^cd^	6.146 ± 0185 ^a^
0.1	9.514 ± 0.144 ^b^	4.652 ± 0.104 ^bc^	14.156 ± 0.245 ^b^	2.045 ± 0.017 ^b^	2.368 ± 0.045 ^b^	5.984 ± 0.113 ^a^
1	10.207 ± 0.091 ^a^	4.836 ± 0.071 ^ab^	15.043 ± 0.160 ^a^	2.111 ± 0.015 ^a^	2.509 ± 0.028 ^a^	5.996 ± 0.102 ^a^
10	8.787 ± 0.336 ^cd^	4.534 ± 0.175 ^c^	13.321 ± 0.508 ^cde^	1.938 ± 0.014 ^c^	2.188 ± 0.100 ^cd^	6.088 ± 0.087 ^a^
50	8.976 ± 0.128 ^c^	5.023 ± 0.093 ^a^	13.999 ± 0.222 ^bc^	1.787 ± 0.008 ^e^	2.271 ± 0.052 ^bc^	6.164 ± 0.047 ^a^
100	8.392 ± 0.071 ^de^	4.537 ± 0.024 ^c^	12.930± 0.095 ^de^	1.850 ± 0.006 ^d^	2.117 ± 0.011 ^de^	6.106 ± 0.052 ^a^
1000	8.167 ± 0.041 ^e^	4.527 ± 0.031 ^c^	12.694 ± 0.069 ^e^	1.804 ± 0.007 ^e^	2.039 ± 0.020 ^e^	6.225 ± 0.077 ^a^
AMX						
0	8.702 ± 0.078 ^b^	4.493 ± 0.038 ^d^	13.195 ± 0.116 ^c^	1.937 ± 0.0015 ^a^	2.295 ± 0.014 ^b^	5.750 ± 0.027 ^d^
0.1	9.166 ± 0.041 ^a^	4.653 ± 0.020 ^c^	13.818 ± 0.058 ^b^	1.970 ± 0.005 ^a^	2.415 ± 0.010 ^a^	5.723 ± 0.012 ^d^
1	9.083 ± 0.256 ^a^	4.913 ± 0.120 ^b^	13.996 ± 0.376 ^ab^	1.849 ± 0.007 ^b^	2.423 ± 0.072 ^a^	5.776 ± 0.016 ^d^
10	9.149 ± 0.079 ^a^	5.149 ± 0.034 ^a^	14.298 ± 0.112 ^a^	1.777 ± 0.004 ^c^	2.411 ± 0.017 ^a^	5.931 ± 0.016 ^bc^
50	8.681 ± 0.040 ^b^	5.006 ± 0.016 ^ab^	13.687 ± 0.028 ^b^	1.734 ± 0.013 ^cd^	2.287 ± 0.013 ^b^	5.985 ± 0.024 ^ab^
100	8.719 ± 0.054 ^b^	5.059 ± 0.015 ^ab^	13.779 ± 0.043 ^b^	1.724 ± 0.015 ^d^	2.288 ± 0.009 ^b^	6.023 ± 0.023 ^a^
1000	8.270 ± 0.097 ^c^	4.737 ± 0.056 ^c^	13.007 ± 0.081 ^c^	1.746 ± 0.037 cd	2.215 ± 0.024 ^b^	5.872 ± 0.034 ^c^
AMP + AMX						
0	8.812 ± 0.041 ^b^	5.129 ± 0.017 ^dc^	13.942 ± 0.053 ^bc^	1.718 ± 0.006 ^a^	2.323 ± 0.016 ^b^	6.002 ± 0.028 ^e^
0.1	9.157 ± 0.019 ^a^	5.501 ± 0.009 ^a^	14.658 ± 0.026 ^a^	1.664 ± 0.002 ^c^	2.406 ± 0.007 ^a^	6.092 ± 0.017 ^d^
1	8.874 ± 0.183 ^b^	5.321 ± 0.104 ^b^	14.195 ± 0.286 ^b^	1.667 ± 0.004 ^c^	2.326 ± 0.047 ^b^	6.103 ± 0.005 ^cd^
10	8.737 ± 0.075 ^bc^	5.292 ± 0.048 ^bc^	14.029 ± 0.122 ^b^	1.651 ± 0.003 ^c^	2.299 ± 0.014 ^b^	6.102 ± 0.019 ^cd^
50	8.790 ± 0.048 ^b^	5.538 ± 0.004 ^a^	14.329 ± 0.051 ^ab^	1.587 ± 0.008 ^d^	2.309 ± 0.016 ^b^	6.206 ± 0.021 ^b^
100	8.135 ± 0.138 ^d^	5.415 ± 0.107 ^ab^	13.550 ± 0.241 ^c^	1.502 ± 0.011 ^e^	2.136 ± 0.038 ^c^	6.343 ± 0.056 ^a^
1000	8.503 ± 0.028 ^c^	5.022 ± 0.028 ^d^	13.526 ± 0.055 ^c^	1.693 ± 0.004 ^b^	2.191 ± 0.006 ^c^	6.174 ± 0.023 ^bc^

Chl*a*—chlorophyll *a*, Chl*b*—chlorophyll *b*, Chl*a* + Chl*b*—chlorophyll *a* + chlorophyll *b*, Car—carotenoids, Chl*a*/Chl*b*—chlorophyll *a*/chlorophyll *b*, Chl(*a* + *b*)/Car—(chlorophyll *a* + chlorophyll *b*)/carotenoids.

**Table 2:** Effect of AMP, AMX, and AMP + AMX mixtures on F_0_, F_v_, F_m_, F_v_/F_m_, and F_v_/F_0_ in wheat seedlings. Values denoted by the same letters in the columns do not differ statistically at *p* < 0.05.

Concentration of Antibiotics (mg·kg^−1^ of Soil DW)	F_o_	F_m_	F_v_	F_v_/F_m_	F_v_/F_o_
**AMP**					
0	198.3 ± 11.6 ^a^	990.0 ± 43.9 ^a^	791.7 ± 33.1 ^a^	0.799 ± 0.004 ^a^	3.995 ± 0.110 ^a^
0.1	195.2 ± 8.8 ^a^	982.3 ± 43.4 ^a^	787.2 ± 35.4 ^a^	0.801 ± 0.003 ^a^	4.034 ± 0.079 ^a^
1	201.3 ± 10.1 ^a^	1012.0 ± 50.2 ^a^	810.7 ± 41.3 ^a^	0.801 ± 0.004 ^a^	4.027 ± 0.110 ^a^
10	197.5 ± 6.3 ^a^	999.2 ± 38.3 ^a^	801.7 ± 33.4 ^a^	0.802 ± 0.005 ^a^	4.059 ± 0.111 ^a^
50	200.3 ± 14.3 ^a^	995.7 ± 75.8 ^a^	795.3 ± 62.1 ^a^	0.798 ± 0.004 ^a^	3.969 ± 0.094 ^a^
100	200.2 ± 11.6 ^a^	970.3 ± 43.6 ^a^	770.2 ± 45.0 ^a^	0.793 ± 0.015 ^a^	3.860 ± 0.330 ^a^
1000	203.2 ± 5.7 ^a^	1025.0 ± 39.2 ^a^	821.8 ± 35.3 ^a^	0.802 ± 0.005 ^a^	4.045 ± 0.133 ^a^
**AMX**					
0	206.5 ± 9.2 ^a^	1021.5 ± 39.2 ^a^	815.0 ± 31.6 ^a^	0.798 ± 0.005 ^a^	3.948 ± 0.108 ^a^
0.1	206.3 ± 7.5 ^a^	1019.0 ± 40.9 ^a^	812.7 ± 34.5 ^a^	0.797 ± 0.004 ^a^	3.938 ± 0.097 ^a^
1	208.2 ± 9.9 ^a^	1033.3 ± 38.3 ^a^	825.2 ± 28.5 ^a^	0.798 ± 0.003 ^a^	3.966 ± 0.057 ^a^
10	202.2 ± 5.8 ^a^	998.8 ± 24.4 ^a^	796.7 ± 20.9 ^a^	0.797 ± 0.005 ^a^	3.958 ± 0.101 ^a^
50	198.3 ± 9.9 ^a^	997.0 ± 30.3 ^a^	798.7 ± 21.2 ^a^	0.801 ± 0.005 ^a^	4.031 ± 0.117 ^a^
100	207.7 ± 7.2 ^a^	1044.3 ± 38.7 ^a^	836.7 ± 31.9 ^a^	0.801 ± 0.002 ^a^	4.028 ± 0.053 ^a^
1000	204.3 ± 10.8 ^a^	1017.7 ± 52.0 ^a^	813.3 ± 41.8 ^a^	0.799 ± 0.003 ^a^	3.981 ± 0.078 ^a^
**AMP + AMX**					
0	204.8 ± 9.2 ^a^	968.7 ± 22.2 ^a^	763.8 ± 15.5 ^a^	0.788 ± 0.006 ^ab^	3.733 ± 0.133 ^ab^
0.1	204.5 ± 8.0 ^a^	961.8 ± 23.3 ^ab^	757.3 ± 17.2 ^ab^	0.787 ± 0.005 ^b^	3.706 ± 0.116 ^b^
1	195.8 ± 5.6 ^ab^	937.7 ± 31.8 ^abc^	741.8 ± 26.5 ^abc^	0.791 ± 0.002 ^ab^	3.787 ± 0.055 ^ab^
10	177.2 ± 20.1 ^bc^	852.0 ± 111.9 ^bc^	674.8 ± 92.4 ^bc^	0.791 ± 0.008 ^ab^	3.803 ± 0.169 ^ab^
50	188.7 ± 12.7 ^abc^	893.5 ± 50.9 ^abc^	704.8 ± 38.5 ^abc^	0.789 ± 0.003 ^ab^	3.738 ± 0.073 ^ab^
100	171.5 ± 9.4 ^c^	841.7 ± 51.5 ^c^	670.2 ± 42.7 ^bc^	0.796 ± 0.004 ^a^	3.907 ± 0.091 ^a^
1000	169.7 ± 17.6 ^c^	833.7 ± 79.0 ^c^	664.0 ± 61.6 ^c^	0.796 ± 0.003 ^a^	3.917 ± 0.079 ^a^

## Data Availability

Data will be made available on request.

## Abbreviations

The following abbreviations are used in this manuscript:

AMP	Ampicillin
AMX	Amoxicillin
ROS	Reactive oxygen species
H_2_O_2_	Hydrogen peroxide
AOX	Complex antioxidant system
MDA	Malondialdehyde
AsA	Ascorbic acid
GSH	Glutathione
GSSG	Oxidized glutathione
SOD	Superoxide dismutase
POD	Guaiacol peroxidase
CAT	Catalase
APX	Ascorbate peroxidase
DW	Dry weight
F_0_	Initial (minimum) fluorescence
F_v_	Fluctuating fluorescence
F_m_	Maximum fluorescence
